# Multi-scale modeling of drug binding kinetics to predict drug efficacy

**DOI:** 10.1007/s00018-019-03376-y

**Published:** 2019-11-25

**Authors:** Fabrizio Clarelli, Jingyi Liang, Antal Martinecz, Ines Heiland, Pia Abel zur Wiesch

**Affiliations:** 1 Department of Pharmacy, Faculty of Health Sciences, UiT The Arctic University of Norway, 9037 Tromsø, Norway; 2 Department of Arctic and Marine Biology, UiT The Arctic University of Norway, 9037 Tromsø, Norway; 3 Centre for Molecular Medicine Norway, Nordic EMBL Partnership, Blindern, P.O. Box 1137, 0318 Oslo, Norway

**Keywords:** Pharmacodynamics, Pharmacokinetics, Binding kinetics, Antimicrobial activity, Mathematical biology, Differential equations

## Abstract

Optimizing drug therapies for any disease requires a solid understanding of pharmacokinetics (the drug concentration at a given time point in different body compartments) and pharmacodynamics (the effect a drug has at a given concentration). Mathematical models are frequently used to infer drug concentrations over time based on infrequent sampling and/or in inaccessible body compartments. Models are also used to translate drug action from in vitro to in vivo conditions or from animal models to human patients. Recently, mathematical models that incorporate drug-target binding and subsequent downstream responses have been shown to advance our understanding and increase predictive power of drug efficacy predictions. We here discuss current approaches of modeling drug binding kinetics that aim at improving model-based drug development in the future. This in turn might aid in reducing the large number of failed clinical trials.

## Introduction

Over the last 50 years, mathematical models describing drug pharmacokinetics (PK) and pharmacodynamics (PD) developed from the first concepts of simple relationships between drug concentration and its effects in the 1960 s [1–5] to advanced models that substantially improve our comprehension of the drug action mechanisms [6–10]. Advances in computational power and the improved accuracy and availability of experimental data have further fuelled model development.

**Fig. 1:**
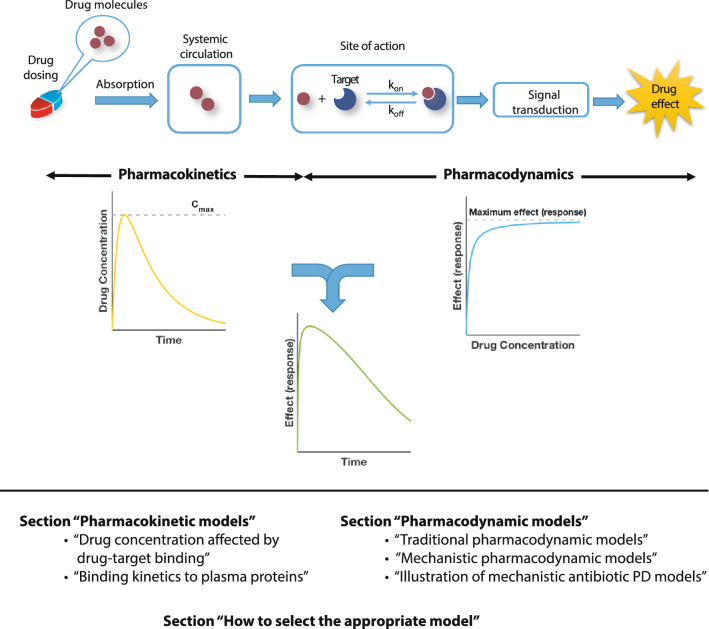
Schematic overview of PK/PD modeling and review outline. The process from drug administration to the emergence of effects consists of PK and PD components. PK describes the drug concentration profile in blood or at the site of action, i.e., the movement of drugs within the body after administration. PD describes how the given drug concentration in its target tissue elicits its effects. Mechanistically, this involves that drug molecules bind to their targets after reaching the desired site of action and induce various signaling transduction pathways, which ultimately, leads to biological effects/responses. Integrated PK/PD models allow us to investigate the drug efficacy over time under different dosing regimens

We discuss mathematical modeling approaches that connect drug binding kinetics with its downstream effects across different scales for a multitude of different applications such as treatments for bacterial and viral diseases, tumors, hypertension and mental illnesses. In this review, we will exemplarily highlight modeling approaches at different scales, starting with pharmacokinetic models including drug-target binding (“Pharmacokinetic models”), over traditional pharmacodynamic models (“Traditional pharmacodynamic models”) to various mechanistic pharmacodynamic drug-target binding models (“Mechanistic pharmacodynamic models”). We will conclude with a guide on how to select appropriate models for the system under investigation (“How to select the appropriate model”). An overview of the approaches discussed here is given in Fig. 1.

This review is not aiming at being a comprehensive description of all possible applications. We instead want to provide an overview of how mathematical models are used to describe PD and PK in a wide range of diseases, and more comprehensively describe PD models of drug-target binding in infectious diseases. Traditional pharmacodynamic models were introduced 50–100 years ago (e.g. the *E*_max_ model and its derivative, the Hill function or sigmoidal *E*_max_ model) [5, 11, 12]. Starting from these models, we discuss the evolution of target binding models and the underlying assumptions that determine in which scenarios those models can be used. At all scales, model complexity can vary considerably depending on the existing knowledge and details required to answer the pharmacological question to be addressed. We describe what can be gained by using more complex modeling approaches and which experimental data need to be available to parametrize those models. Of note, while the models we focus on describe antibacterial action, many of them may be applicable to other scenarios, for example tumor cells.

### Pharmacokinetics and pharmacodynamics

PK describes the drug concentration profile in blood or at the site of action, i.e., the movement of drugs within the body after administration. PD describes how the given drug concentration in its target tissue elicits its effects. While multi-scale models that describe both PK and PD in mechanistic detail have been developed [13], we discuss PK and PD approaches separately for clarity.

PK describes the “movement” of the drug in the human body, often subdivided in compartments. The different drug concentrations in different body compartments are governed by absorption, distribution, metabolism and elimination or excretion [14]. As soon as the drug enters the bloodstream, it can potentially be delivered to the site of action. Often, the target tissue in which the drug action occurs is inaccessible or not practical for routinely measuring local concentrations. Reliable estimates of the relationship between drug concentration in plasma and the target tissue are difficult to obtain and typically involve sophisticated models. While a detailed PK model has been successfully applied to tuberculosis to describe local drug concentrations in the target tissue [10], the data used to validate the model came from an extremely invasive procedure (lung resection). As a result, data on local drug concentrations are rarely available and it is therefore estimated by other means, such as using a linear relationship.

Once the drug reaches the site of action, PD describes the relationship between drug concentration and its efficacy. It is important to decide which effect we are interested in, and how this is related to target occupancy. In some cases, such as virus neutralization by antibodies, the observable endpoint that defines efficacy can be easily ascribed to molecular mechanisms. Direct drug efficacy can be predicted by solely using binding kinetic models. In other therapeutic areas, the observable effects are very complex, such as behavioral changes in mental illnesses. These complex processes are presumed to be related to target occupancy, which in turn leads to an inhibition or activation of downstream signaling pathways. Indirect drug efficacy is therefore mathematically described as a function of the amount of bound target, *E* = *f*(*AT*). This function can be either completely mechanistic when all contributing molecular pathways are known, or only partly mechanistic, such as when modeling drug-target binding and subsuming downstream mechanisms in a dose–response function.

Sometimes, the boundaries between modeling PK and PD are not well defined. It may, for example, depend on whether one defines an entire bacterium or the molecules that bind to the antibiotic inside the bacterial cell as the “target”. In the former case, diffusion across the bacterial cell envelope would be described by a pharmacodynamic model, and in the latter by a pharmacokinetic model. For the purpose of this review, we define mathematical models describing the drug penetration in a host’s target tissue as pharmacokinetics describing drug penetration into foreign organisms (e.g. bacteria) as pharmacodynamics, even though this distinction is somewhat arbitrary. Ultimately, these distinctions may be of secondary importance since the mathematical terms describing drug penetration into a body compartment or a bacterial cell can be part of either PK and PD model and both together are used to predict a drug’s effect-time profile for various dosing regimens (Fig. 1).

### Binding kinetics

For our purposes, drug binding kinetics describes the interactions between a drug molecule *A* (ligand) and a specific or unspecific binding partner *B* (target or receptor), which form the ligand–receptor complex *AB*: $A + B\mathop {\mathop \rightleftharpoons \limits^{{k_{\text{on}} }} }\limits_{{k_{\text{off}} }} AB$. In this reaction, *k*_on_ (units M^−1^ s^−1^) is the association (binding) rate and *k*_off_ (units s^−1^) is the dissociation rate per second. The dissociation constant *K*_D_ (unit M, M = mol per liter) is defined as $K_{\text{D}} = \frac{{k_{\text{off}} }}{{k_{\text{on}} }}$. It describes the molar concentration at which half of the total binding partner molecules are occupied at equilibrium and is a measure of the binding affinity. The half-life of the complex *AB* is given by $t_{1/2} = \frac{\ln (2)}{{k_{\text{off}} }}$.

## Pharmacokinetic models

Pharmacokinetic models are mostly used in the pre-clinical and clinical drug development to calculate required drug concentrations and treatment schedules. They usually describe drug absorption, distribution, processing and elimination but not the molecular mechanisms at the drug target site. Drug binding can affect local and global drug concentrations in various ways.

### Drug concentration affected by drug-target binding

**Fig. 2:**
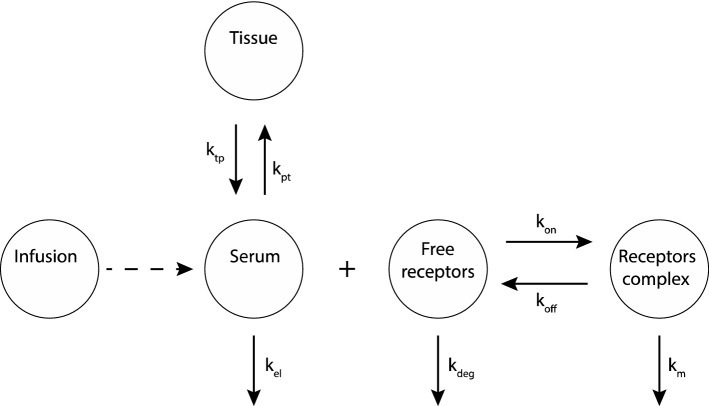
PK/PD model. The drug can be infused in the central compartment (Serum) and it can bind to non-specific targets in the tissue compartment. Also, the drug can be eliminated from the central compartment with a rate *k*_el_, or bind to free targets (free receptors) to form a drug-target complex (receptor complex) with an association rate *k*_on_ and a dissociation rate *k*_off_. The complex can degrade with a rate *k*_m_, while free receptors can degrade with a rate *k*_deg_. Modified from [15]

Mager and Sugiyama [15–18], combined PK and PD models to describe drugs that bind with high affinity, using the term “Target Mediated Drug Disposition” (TMDD, introduced by Levy [19]). TMDD refers to drug molecules that “bind with high affinity” to their targets [15], and a substantial amount of target is present and may exhibit a nonlinear PK behavior. To describe such behavior, the researchers introduced a two-compartment PK model with a PD target binding model [15]. The drug concentration in the central compartment (*C*_p_) binds (*k*_on_) with its targets to form a complex (DR) that has a total binding capacity *R*_max_. The complex DR can dissociate (*k*_off_) and degrade with a rate constant, *k*_m_. In the central compartment (Serum), the drug can be eliminated (*k*_el_) or can bind with non-specific targets in tissue (DT), see Fig. 2. Here, targets are assumed to be homogeneous, equivalent and independent. Traditional pharmacodynamic approaches (Hill-functions) are used to describe drug efficacy of the resulting local concentrations. The models consist of systems of ordinary differential equations, and they are used (with appropriate adjustments) to describe several examples like angiotensin-converting-enzyme inhibitor (ACE inhibitor), imirestat, warfarin, bosentan, monoamine oxidase type B inhibitors and natalizumab [17].

### Binding kinetics to plasma proteins

Drugs taken up through the gastrointestinal system are transported in the blood. It should be noted that many drugs in the blood are bound to transport proteins, which is strongly influencing their free and thus effective concentration. As blood transport proteins un-specifically bind to small molecules, several drugs applied in combination, competitively affect their respective binding and release and thus the effective drug concentration. The effective drug concentration is often approximated as a constant percentage of plasma protein binding. However, this can be highly misleading as drug binding to either the targets or unspecific molecules decreases the free drug concentration [20]. The lower free drug concentration, in turn, shifts the equilibrium of drug-plasma protein binding and leads to a release of drug. Depending on the drug, modeling the kinetics of protein plasma binding can, therefore, be crucial [21]. As, furthermore, nutritional components as well as other drugs administered at the same time can be bound by the same transport proteins, other drugs as well as nutrition can affect free drug concentration and thus efficacy [22].

## Pharmacodynamic models

### Traditional pharmacodynamic models

**Fig. 3:**
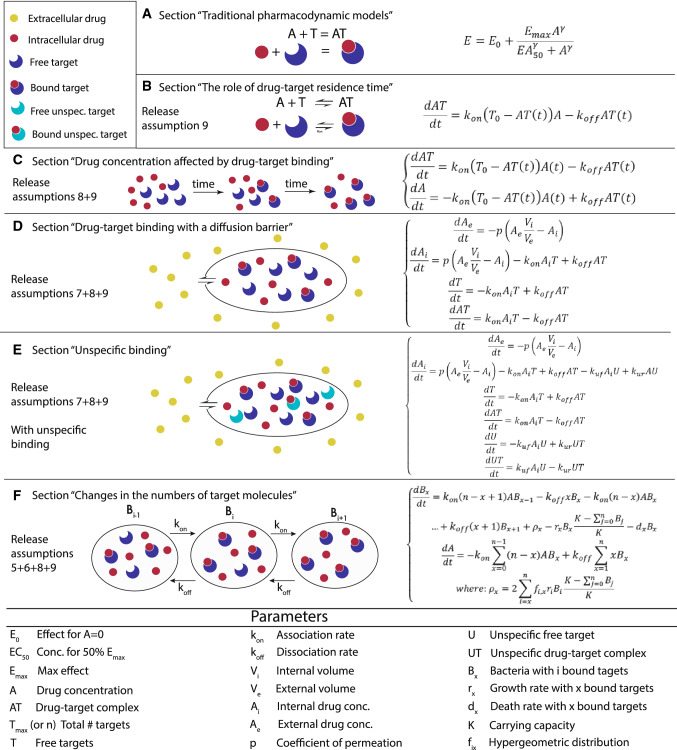
Overview of the main developments in modeling PD. In this figure, we give an overview of modeling approaches with increasing complexity that are commonly used to describe the molecular mechanisms of anti-infective drugs. The left column refers to the respective section in the text that describes the model as well a list of assumptions that are relaxed compared to a traditional Hill function. A drawing in the central column illustrates the model. Finally, the column on the right gives the respective equations. The parameters are described in the table below. In **a**, we describe the reaction at the equilibrium (*E*_max_, Hill), i.e., the time to reach the equilibrium of the reaction is neglected (“Traditional pharmacodynamic models”). In **b**, we relax the assumption 9 of the instantaneous equilibrium, i.e., we have the complete kinetic equation to describe the variation in time of the drug-target complex (see “The role of drug-target residence time”). In **c**, we release assumption 8 of constant drug concentrations, i.e., we add an equation describing a variable drug concentration. This can be a PK model added to our binding kinetics equation (“Drug concentration affected by drug-target binding”). In **d**, we release assumption 7. In this way, the internal drug concentration is not equal to the external drug concentration, i.e., we need an equation for the external and one for the internal drug concentration (“Drug-target binding with a diffusion barrier”). In **e**, we add unspecific targets with their own equations, and drug molecules can associate/dissociate to unspecific targets. Thus, fewer drug molecules are available for drug action (“Unspecific binding”). In **f**, we introduce the replication and death of bacteria, which leads to changes in the number of target molecules, i.e., we relax assumption 4 and 5, but not 6 and 7. To do so, bacteria are classified in compartments according to the number of bound target molecules. Each compartment is described by separate equation. (“Changes in the numbers of target molecules”)

Traditional PD models, i.e., *E*_max_ or Hill-functions can be derived using simplifying assumptions based upon the kinetics of drug-target binding. The effect of a drug at an observed site is related to the drug’s concentration and time since administration. The relationship between the drug concentration and its effect is not linear [5], and the Hill function has been used to describe this relationship. The model is based on the idea that under certain assumptions (discussed below and in Fig. 3), the rate of change of the drug-target complex AT is given by the following equation:

1 $$ \frac{{{\text{d}}AT}}{{{\text{d}}t}} = k_{\text{on}} \left( {T_{0} - AT\left( t \right)} \right)A - k_{\text{off}} AT\left( t \right), $$

where *k*_on_ is the association rate, *k*_off_ is the dissociation rate, *T*_0_ is the initial number of targets and *A* is the drug concentration. At the equilibrium, Eq. (1) becomes

2 $$ AT = \frac{{T_{0} A}}{{k_{\text{D}} + A}}, $$

where *K*_D_ = *k*_off_/*k*_on_.

In its simplest form, *E*_max_ models are proportional to the concentration of the drug-target complex *AT*: $E = E_{0} + \frac{{E_{\hbox{max} } A}}{{{\text{EC}}_{50} + A}}$. Here, *E* is the effect of the drug at a given concentration of drug *A*, *E*_max_ is the maximum effect, EC_50_ is the concentration of drug that produces the 50% of *E*_max_ and *E*_0_ is the effect when *A* = 0 (additional details in [1]). In 1910, Hill investigated the shape of the oxygen–hemoglobin saturation relationship [11, 23]. The shape of the curve was steeper than the predictions obtained with the steady-state solution (Eq. 2). To solve this issue, Hill added an exponential parameter to the model, the Hill exponent $\gamma$ [23]. Thus, Eq. (2) can be easily transformed into the Hill function (Eq. (3) and Eq. A in Fig. 3):

3 $$ E = E_{0} + \frac{{E_{\hbox{max} } A^{\gamma } }}{{{\text{EC}}_{50}^{\gamma } + A^{\gamma } }}. $$

For drugs that show a steeper relationship between drug exposure and effect than the predictions with the *E*_max_ model, a Hill-function (also called sigmoidal *E*_max_ model, even though simple *E*_max_ models are also sigmoidal) can be used. The simplicity of *E*_max_ models results in their frequent application when little is known about the mechanisms of action for a given drug. However, it is important to realize that these models rely on a large number of assumptions (many of which are likely invalid in reality):

1. The number of target molecules is constant. In the case of intracellular targets, the number of targets per cell, i.e., the concentration is constant. If binding is modeled as a multistep process to a macromolecule, the number of binding sites per macromolecule is constant.
2. Independence of receptors. The probability of a receptor to bind a drug molecule is not affected by the number of other bound complexes. This implies that it is not important in which order they bind.
3. The distribution of receptors is homogeneous. The intracellular space and diffusion are not included in these models, and for this reason, each receptor has the same probability of contact and therefore binding with the ligand.
4. Reversible binding has biologically reversible consequences. This assumption is violated when cells, such as bacteria or cancer cells, die due to the effect of bound targets. Even if the drug afterwards dissociates from its target, the cell death is irreversible.
5. No changes in the number of target molecules in the entire system, e.g., in a bacterial population or in a tumor. This precludes cellular growth or death.
6. All targets or receptors are equivalent. This means that all the targets that bind the drug with the same rate elicit the same response to the drug.
7. The time required to pass through the cell envelope is negligible. Under this assumption, the drug concentration outside the cell is equal to the drug concentration inside the cell.
8. The drug concentration is constant during the time required to reach the equilibrium. This ensures that we can consider only the steady-state for each value of drug concentration, and it is sufficient to know only the rate *k*_D_ = *k*_off_/*k*_on_ (see Eq. 2).
9. The rates of drug-target association and dissociation are very fast. Since the equilibrium happens when the derivative is zero (no more variation in time), one of the assumptions is that the association and the dissociation rates have to be fast enough so that the time to reach the equilibrium can be neglected. It is important to remember that, if the reaction rates are not fast enough, we need to use the full Eq. (1) instead of using only its equilibrium approximation in Eq. (2).

### Mechanistic pharmacodynamic models

To overcome the limitations of empirical PD models, more and more detailed mechanistic models of drug binding and response have been developed in recent years. We start out with discussing approaches of increasing complexity that describe drug-target binding

#### Modeling drug-target binding

The use of mechanistic PD models for describing antibacterial action was pioneered by Hedges in 1966 [24]. In this work, the authors describe the adsorption kinetics of a lethal amount of the bacterial toxin colicin by bacteria (e.g. *E. coli*). In this model, by analogy with photons in radiation, the targets are hit (and not bound) by the drug molecules. The model includes assumptions 1–9, which are listed above. The model describes two phases, in the first one the colicin is absorbed by receptors, and, in the second one, the lethal effect happens after the completion of phase 1 [4]. Finally, the lethal effect is defined once *r* targets (the threshold) are occupied (*r* < *n*).

Since then, models of increasing complexity have been employed. Figure 3 summarizes the main developments in the modeling of drug-target binding kinetics, starting from traditional Hill functions and successively relaxing assumptions, culminating with the equations describing the entire population dynamics of bacteria in response to drug-target binding.

##### The role of drug-target residence time

Both traditional PD models and the earliest mechanistic PD model by Hedges [24] assume that the binding equilibrium is instantaneously reached. This makes the implicit assumption that only the equilibrium constant *K*_D_ = *k*_off_/*k*_on_ determines drug efficacy. The magnitude of the association rate *k*_on_ and dissociation rate *k*_off,_ and thereby binding kinetics, is assumed to have negligible influence. This has been challenged by Copeland et al. [25], who develop a concept from Ehrlich [26]: “a drug is efficacious only so long as it is bound to, and modulating the action of, its physiological target(s)”. The focus is, therefore on the crucial role played by the drug-target complex. In other words, the residence time (*t*_red_ = 1/*k*_off_, which depends on the dissociation rate) of the drug-target complex is more important than the simple affinity. For the authors (see [25, 27]), the dissociation rate *k*_off_ is more important than *k*_on_, due to several constraints acting on *k*_on_ (it can range considerably), while the dissociation rate *k*_off_ is entirely dependent on the reaction kinetics between the drug molecules and their targets. They, therefore argue that the optimization of the dissociation rate is of primary importance. The authors applied this modeling concept to viruses, inhibitors of steroid 5β-reductase, inhibitors of purine nucleoside phosphorylase (PNP) and angiotensin II type 1 receptor (ATR1). Other interesting studies modeling the kinetics of drug-target binding rather than instantenous equilibria include Tonge [28], Shimada [29] and Walkup [30].

##### Models describing multimer targets

**Fig. 4:**
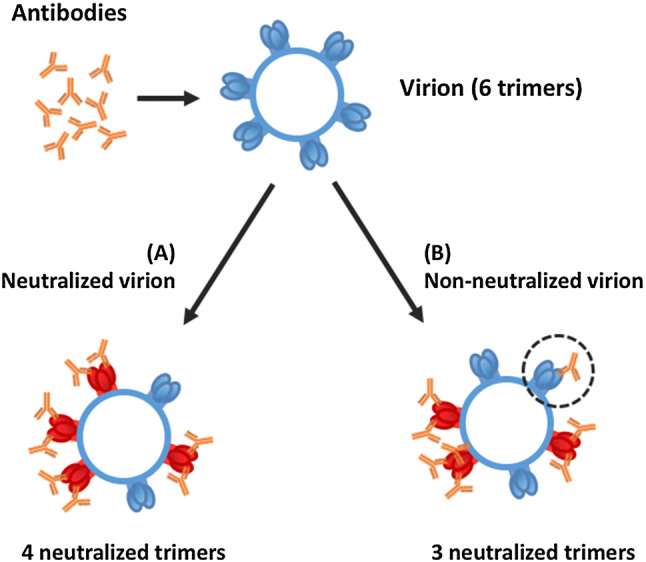
Virion neutralization. An example of how random antibody binding can have different effects. Here, eight antibodies bind in two different ways to a virion with six trimers. In this example, the minimum number of bound trimers to neutralize a virion is four with at least two antibodies each. In (A), the virion is neutralized, four different trimers are bound to two antibodies. In (B), one trimer is bound to three antibodies and one trimer is bound with only one antibody (dashed circle), then there are only three trimers neutralized. Modified from [31]

The majority of models assume that the target is one molecule with one single binding site. Many drug targets are homo- or hetero-multimers with multiple binding sites. One example is homo-trimeric spikes on the surface of HIV virions that enable the virion to enter host cells. This has been modeled by Magnus et al. [31–33]: the authors estimate the number of antibodies (called stoichiometries) required for neutralizing a single virion and a whole virion population. The number of (HIV) spikes necessary for cell entry, combined with the minimal number of antibodies able to neutralize one spike (or trimer), permits to estimate how many antibodies are needed to neutralize a single virion and an entire population of virions. However, this estimation is not trivial as Fig. 4 illustrates: if there are more binding sites per trimer than needed for neutralization, a substantial fraction of antibodies will bind “unnecessarily” to already neutralized trimers, thereby reducing efficacy.

Here, the assumption of equivalent receptors (assumption 6 on the list in “Traditional pharmacodynamic models”) is invalid, because the same number of bound targets, combined differently, can have different outcomes.

##### Drug-target binding with a diffusion barrier

In some cases, we need to extend the model to include the diffusion throughout the bacterial cell envelope for a variable drug concentration. Notably, the influx and efflux of drug molecules across the cell envelope are regulated by multiple mechanisms, which depends on the structure of the cell envelope and characteristics of drug molecules. Mathematical models on how drug molecules cross the cell envelope have been developed with different complexity levels (e.g. [7, 34]). However, the modeling approach can be limited due to lack of knowledge on the specific mechanisms. Assuming that the drug molecules only enter into the cell by diffusion, as shown in Fig. 3d (passive diffusion), we consider drug molecules outside bacterial cells that need to traverse the cell membrane by diffusion.

4 $$ \begin{aligned} & \frac{{{\text{d}}A_{\text{e}} }}{{{\text{d}}t}} = - p\left( {A_{\text{e}} \frac{{V_{\text{i}} }}{{V_{\text{e}} }} - A_{\text{i}} } \right) \\ & \frac{{{\text{d}}A_{i} }}{{{\text{d}}t}} = p\left( {A_{\text{e}} \frac{{V_{\text{i}} }}{{V_{\text{e}} }} - A_{\text{i}} } \right) - k_{\text{on}} A_{\text{i}} T + k_{\text{off}} AT \\ & \frac{{{\text{d}}T}}{{{\text{d}}t}} = - k_{\text{on}} A_{\text{i}} T + k_{\text{off}} AT \\ & \frac{{{\text{d}}AT}}{{{\text{d}}t}} = k_{\text{on}} A_{\text{i}} T - k_{\text{off}} AT. \\ \end{aligned} $$

Here, *A*_e_ is the external number of antibiotic molecules, *A*_i_ is the intracellular number of antibiotic molecules, *T* is the number of free targets, *AT* is the number of drug-target complexes, *p* is proportional to the permeability.

##### Unspecific binding

An additional step involves the inclusion of unspecific binding by adding terms that describe how a drug *A* binds to an unspecific binding site *U* to form an unspecific complex *AU* (see Fig. 3e). Unspecific binding partners are often assumed to be ubiquitous, such that binding never saturates and therefore the number of free binding sites *U* does not change (i.e., does not need to be modeled explicitly). The unspecific binding rate is denoted as *k*_u,f_ and the unspecific dissociation rate as *k*_u,r_. This model can be expressed as follows [7]:

5 $$ \begin{aligned} & \frac{{dA_{e} }}{dt} = - p\left( {A_{e} \frac{{V_{i} }}{{V_{e} }} - A_{i} } \right) \\ & \frac{{{\text{d}}A_{i} }}{{{\text{d}}t}} = p\left( {A_{\text{e}} \frac{{V_{\text{i}} }}{{V_{\text{e}} }} - A_{\text{i}} } \right) - k_{\text{on}} A_{\text{i}} T + k_{\text{off}} AT - k_{\text{uf}} A_{\text{i}} U + k_{\text{ur}} AU \\ & \frac{{{\text{d}}T}}{{{\text{d}}t}} = - k_{\text{on}} A_{\text{i}} T + k_{\text{off}} AT \\ & \frac{{{\text{d}}AT}}{{{\text{d}}t}} = k_{\text{on}} A_{\text{i}} T - k_{\text{off}} AT \\ & \frac{{{\text{d}}U}}{{{\text{d}}t}} = - k_{\text{uf}} A_{\text{i}} U + k_{\text{ur}} UT \\ & \frac{{{\text{d}}UT}}{{{\text{d}}t}} = k_{\text{uf}} A_{\text{i}} U - k_{\text{ur}} UT. \\ \end{aligned} $$

##### Changes in the numbers of target molecules

For antibiotics and anti-cancer drugs, the number of targets can change over time, because cancer cells or bacteria replicate (violating assumption 5). At the same time, bacteria or cancer cells die due to the effect of bound targets. This violates assumption 4, i.e., that a chemically reversible process is also biologically reversible: Let us assume that n bound targets kill a bacterial or cancer cell and that exactly *n* targets are currently bound to a dead cell. One drug-target complex may dissociate such that only *n *− 1 targets are bound, but this will not lead to a resurrection of the dead cell. Thus, if the desired pharmacological effect means minimizing a cell population and that cell population replicates and dies, the binding kinetics will be affected by the dynamics of that cell population. This can only be neglected when the action of the drug is sufficiently fast such that the cells neither replicate nor die until the chemical reaction has reached equilibrium.

To incorporate both bacterial growth and death into the model, living bacteria can be classified into compartments based on the number of bound target molecules *x* [6], out of a total of *n* target molecules per bacterium. Here, bacterial cells with *n* target molecules are equivalent to molecules with *n* independent binding sites. The association and dissociation of the target and antibiotic molecules are described by the following system of differential equations.

6 $$  \begin{array}{llll} {\frac{{{\text{d}}B_{x} }}{{{\text{d}}t}} = k_{\text{on}} \left( {n - x - 1} \right)AB_{x - 1} - k_{\text{off}} xB_{x} - k_{\text{on}} \left( {n - x} \right)AB_{x} + k_{\text{off}} \left( {x + 1} \right)B_{x + 1} + \rho_{x} - r_{x} B_{x} \frac{{K - \mathop \sum \nolimits_{j = 0}^{n} B_{j} }}{K} - d_{x} B_{x} } \\ {\frac{{{\text{d}}A}}{{{\text{d}}t}} = - k_{\text{on}} \mathop \sum \limits_{x = 0}^{n - 1} \left( {n - x} \right)AB_{x} + k_{\text{off}} \mathop \sum \limits_{x = 1}^{n} xB_{x} } \\ \rho_{x} = 2\mathop \sum \limits_{i = x}^{n} f_{i,x} r_{i} B_{i} \frac{{K - \mathop \sum \nolimits_{j = 0}^{n} B_{j} }}{K}, \\ \end{array}   $$

where living bacteria replicate at a rate *r*_*x*_, a function of the number of bound target molecules *x*, as well as they die at a rate *d*_*x*_, a function of the bound targets *x*. *K* is the carrying capacity of the total bacterial population.

The number of targets per bacterium is constant, i.e., it doubles when bacteria duplicate, but the number *x* of bound targets in the mother cells remains constant during the duplication and it is distributed in the two daughter cells following the hypergeometric distribution *f*_*i,x*_ (see Fig. 3f).

#### Linking target occupancy to drug efficacy

Drug efficacy is the capacity of a drug to produce an effect after binding to its target. Various measures are used to quantitatively evaluate the drug efficacy in in vitro, ex vivo and in vivo studies. Binding kinetics and the residence time of the drug-target complex gain more and more attention and are recognized as reliable indicators for drug efficacy. The traditional PK/PD approach to predict drug efficacy by correlating an observable drug effect to measures of drug exposure such as peak concentration (*C*_max_) or average concentration (area under the curve, AUC) is based on assumptions of a rapid equilibrium between the free and bound drug (Fig. 3a). PK/PD models that incorporate the complete kinetic scheme of drug-target binding (Fig. 3b–f) enable us to predict drug efficacy from target occupancy under non-equilibrium conditions, which is more likely to occur in an open systems such as the human body [19]. For drugs that slowly dissociate from their targets, the free drug and drug-target will not be in rapid equilibrium. In this case, the traditional PK/PD model may underpredict the drug effect [22], whereas models with time-dependent drug-target binding will be more mechanistic and suitable for analyzing the relationship between drug concentration and efficacy [21].

However, it is challenging to estimate the relationship (i.e., define the function effect *E* = *f*(*AT*), see “Pharmacokinetics and pharmacodynamics”) between target occupancy and drug effects. Here we summarize models incorporating a detailed kinetic scheme of drug-target binding and a function *f* for various diseases in Table 1.

**Table 1:** Mathematical models with different drug efficacy functions by which drug efficacy is defined as a function of the target occupancy

Function type	Drug (*A*)	Target (*T*)	Effect (*E*)	Function description	References
Linear function	Calcium channel antagonists	Calcium channel	Antihypertensive effect	$\frac{{{\text{d}}E}}{{{\text{d}}t}} = k_{\text{on}} A\left( {E_{ \hbox{max} } - E} \right) - k_{\text{off}} E$	[29]
Linear function	Two drugs: aspirin and NSAIDs (e.g. ibuprofen ($A_{\text{ibu}}$))	Platelet cyclooxygenase-1	Inhibition on platelet aggregation (aspirin: irreversible acetylation; ibuprofen: reversible inhibition)	$E = \left( {T + A_{\text{ibu}} T} \right)\left( {1 - \alpha A_{\text{ibu}} } \right)$ $\alpha$: coefficient for ibuprofen efficacy	[35]
Linear function	Buprenorphine	μ-Opioid receptor in the brain	Respiratory depressant and antinociceptive effects	Respiratory depressant effect $E = E_{\text{baseline}} \left( {1 - \alpha \rho_{\text{app}} } \right)$ Antinociceptive effects $E = \frac{{E_{\text{baseline}} }}{{1 - \rho_{\text{app}} }}$ $\rho_{\text{app}}$: apparent fractional receptor occupancy $E_{0}$: baseline response $\alpha$: intrinsic activity of the drug	[36]
Linear function (with condition)	Translation inhibiting antibiotics (e.g. tetracycline)	Ribosomes	Bacteriostatic action	Growth rate of bacteria is $$ r_{\text{growth}} \left( {f_{\text{c}} } \right) = \left\{ {\begin{array}{*{20}c} {0 \quad f_{\text{free}} < f_{\text{c}} } \\ {\frac{1}{{1 - f_{\text{c}} }}f_{\text{free}} - f_{\text{c}} \quad f_{\text{free}} > f_{\text{c}} } \\ \end{array} } \right. $$ $f_{\text{free}}$: the fraction of unbound drug $f_{\text{c}}$: critical threshold	[27]
Linear function (with condition)	LpxC inhibitors	LpxC enzyme	Killing of *Pseudomonas aeruginosa*	The killing rate of bacteria $k_{\text{kill}} \cdot AT$($k_{\text{kill}}$ is the maximum killing rate constant) when $AT$ is between the minimum and maximum target occupancy required for the antibacterial effects	[22]
Sigmoid function (Hill equation)	Antipsychotic drugs	Dopamine D_2_ receptor	Cellular cAMP response for competition binding between antagonists ($A_{\text{a}}$) and dopamine ($A_{\text{d}}$) to D_2_ receptor	Overall effect on the rate of cAMP production by receptor antagonists and dopamine is $\left( {k_{0} + K_{\hbox{max} } \frac{{(A_{\text{a}} T)^{n} }}{{(A_{\text{a}} T_{50})^{n} + (A_{\text{a}} T)^{n} }}} \right)\left( {1 - \frac{{(A_{\text{d}} T)^{n} }}{{(A_{\text{d}} T_{50})^{n} + (A_{\text{d}} T)^{n} }}} \right)$	[37]
Sigmoid function (Hill equation)	Trastuzumab-valine-citrulline-monomethyl auristatin E	Tubulin	Killing of tumor cell	Killing rate $r\left( {AT} \right) = K_{\hbox{max} } \frac{{AT^{n} }}{AT^{n}_{50} + AT^{n}}$	[38]

The simplest approach (and the implicit assumption in most PD models) is to assume a linear relationship between target occupancy and drug effects. For example, models of calcium channel blocker in essential hypertension patients [29], gastric acid secretion in dogs [39], antiplatelet effects of aspirin and ibuprofen in human [35] and inhibition of DPP-4 activity in patients with type 2 diabetes [40], use positive or negative linear functions to convert the level of drug-target complex to the corresponding pharmacological responses. When the total number of targets at the site of action is uncertain or impossible to be measured, apparent fractional receptor occupancy can be used by setting the receptor level to one unit [36]. Some models define a linear correlation with certain conditions. In a model of paLpxC inhibitor in animal studies of *Pseudomonas* infection, Walkup et al. [30] assume a saturation limit of the drug-induced killing of bacteria that the killing rate is linearly increased to target occupancy between the minimum and maximum target occupancy required for the antibacterial effects. Similarly, we earlier defined [6] that the replication rate of bacteria linearly depends on the uninhibited ribosomes above a critical threshold.

For some cases, experimental evidence also suggests a nonlinear relationship between observed receptor occupancies and effects [41], and several models that use sigmoid functions to define the relationship between the level of drug-target complex and the pharmacological effects [37, 38]. For instance, a recent in vitro and in silico combined model consists of competitive binding between D_2_ receptor antagonist and endogenous dopamine as well as the downstream response of cellular cyclic adenosine monophosphate (cAMP) [37]. The production rate of cAMP is oppositely affected by the concentration of D_2_-receptor-antagonist complex and receptor–dopamine complex, using a combination of Hill equations.

Alternatively, PD/PK models that incorporate explicit mechanistic simulation of downstream processes initiated by drug- target binding is possible. With increasing knowledge about the molecular mechanisms of diseases, we can develop increasingly complex models of intracellular drug responses. This approach is especially useful when the direct relationship between target occupancy and response is complicated and uncertain. Models demonstrate that the binding of drug-target can induce downstream signal transduction and feedback mechanisms, therefore, affect the drug efficacy at a network level [42].

### Illustration of mechanistic antibiotic PD models

**Fig. 5:**
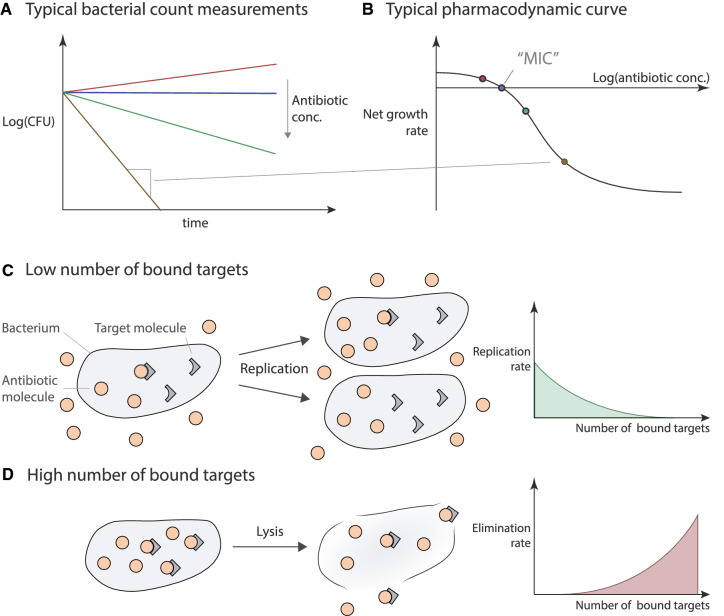
Illustration of the modeled antibiotic action—**a** illustrates typically measured bacterial count measurements over time. These measurements are often used to create “pharmacodynamic curves” (**b**) that define the relationship between the net growth of bacteria (the total replication minus death) at a given antibiotic concentration. In this framework, the minimal inhibitory concentration (MIC) is the concentration when the net growth rate predicted by pharmacodynamic curve is zero. In drug-target binding models, the number of bound targets (at a given intracellular antibiotic concentration) affects either bacterial replication, death, or both (**c**, **d**). **c** Illustrates the replication part, in addition, it also illustrates the assumption that during this process the bound targets are distributed randomly among daughter cells [6]. **d** Illustrates the bacterial elimination part, it also illustrates the possibility that upon elimination, bacteria lyse and release their contents into the extracellular space. This, in turn, may eventually reduce the free antibiotic concentrations

Figure 5 illustrates the observable endpoints of antibiotic efficacy in vitro and crucial aspects of antibiotic action that can be described by mechanistic PD models.

The aim of antibiotic treatment is to reduce the number of bacteria in a patient’s body. The efficacy of antibiotics to do so is typically assessed by so-called time-kill curves (Fig. 5a), where bacterial counts are measured over time after exposure to antibiotics at increasing concentrations. These can be used to estimate the PD curves for a given drug-bacteria combination in a given environment (Fig. 5b). It is important to note that PD curves measure the net population change rather than the replication (Fig. 5c) and death of bacteria (Fig. 5d) separately. As a result, a zero-net change in the population size at the minimal concentration at which bacterial growth stops, i.e., when the net change in the population is exactly zero (minimal inhibitory concentration or MIC) does not necessarily mean that there is no replication of bacteria in a given sample, a very high replication and death rate that neutralize each other would yield the same observation. Figure 5c, d also demonstrate mechanisms that can alter the effects of antibiotic action: upon the replication of bacteria, the bound targets in cells are distributed (randomly) among the daughter cells. If drug-target association and dissociation are on a comparable timescale to replication rates, this can affect the efficiency of the given antibiotic (“Changes in the numbers of target molecules”). Furthermore, after a cell dies, its targets do not immediately disappear, and these targets can leak into the extracellular space. Therefore, even replication and death can eventually lower the free extracellular antibiotic concentrations, which in turn can affect the antibiotic action on other cells.

## How to select the appropriate model

Traditional PD models have substantial advantages, such as their simplicity and the fact that a relatively moderate amount of experimental data is needed to parametrize them. However, traditional PD models are typically unable to capture a variety of PD effects. When such effects are observed, an explicit, mechanistic model may be a better choice because “tweaking” traditional approaches may result in equally complex mathematical models that, in addition to their complexity, have no mechanistic basis and are challenging to parametrize with experimental data. Table 2 provides an overview of PD effects (with a focus on antibiotics, but similar effects have been observed in other systems) that can be easily described using mechanistic models.

**Table 2:** Summary of effects explained by the models in the last column

Effect	Description	Models including this effect
Post-antibiotic effect (PAE)	PAE is a delayed bacterial regrowth after antibiotic exposure	[6, 7, 30]
Inoculum effect	The efficacy of a drug concentration can be a function of the initial bacterial concentration. Increased initial bacterial concentration can imply decreased antibiotic efficacy	[6, 8, 43, 44]
Heterogeneous population	The bacterial population can be heterogeneous, i.e. with different values in: (a) Minimum inhibitory concentration (b) Minimum bactericidal concentration (c) Total number of target molecules (d) Permeability of the cell membrane (e) Minimum threshold of bound targets to kill bacteria (f) Growth rate as a function of bound targets (g) Death rate as a function of bound targets. All of these parameters and functions are important in the efficacy of the drug.	[6]
Off-target binding	Unspecific targets that can bind to the drug molecules. This implies that the total amount of drug molecules available to bind to main targets (action of the drug) can be smaller	[6]
Synergistic and antagonistic action of drugs	Loewe additivity and Bliss independence are compared. Bliss independence is suggested in the presence of different targets. Loewe additivity when the antimicrobials target the same component	[9]

### Post-antibiotic effect

Sometimes, after antibiotic exposure, bacterial regrowth is delayed. This is called “post-antibiotic effect” (PAE). The reason is that the drug-target complex requires some time to dissociate and free the targets, as well as the drug molecules to leave the intracellular space. To explain this effect, we need models with explicit association and dissociation terms, but also a growth rate and a death rate as a function of the number of bound targets are helpful. Models with the association and dissociation rates can explain this effect as demonstrated by Walkup [30] and Abel zur Wiesch [6, 7].

### Inoculum effect

A second challenge is to determine reliable drug dosing able to clear an infection. Predictions for optimal drug concentrations can be distorted by the initial bacterial concentration, i.e., increased initial bacterial concentration can imply a decreased antibiotic efficacy [43]. A useful review of the inoculum effect in vivo and in vitro with beta-lactams (where this effect is pronounced) is given in [44]. One explanation for this effect relates to the fact that the change in the drug availability after binding can be a function of the drug affinity. This effect can be explained by a kinetic model of binding—intuitively, drug molecules that are already bound to their targets cannot kill more bacteria [6, 8].

### Heterogeneous population

In reality, the bacterial population can be heterogeneous. Several parameters can change, and this has consequences on the behavior of the bacterial population. For example, parameters can include the minimum inhibitory concentration or the minimum bactericidal concentration, the total number of target molecules, the permeability of the cell membrane, the minimum threshold of bound targets to kill bacteria and the growth rate and the death rates described as a function of the number of bound targets. All of these parameters and functions are important in determining the efficacy of a drug and can be easily incorporated into mechanistic models [6] by using distributions rather than fixed parameter values to inform a model.

### Synergistic and antagonistic action of drugs

It is crucial to determine when multiple antibiotics can have synergistic or antagonistic effects. Understanding how bacterial populations react to multi-drug treatment can be surprisingly complex. In particular, it is unclear what the “null-hypothesis” for an independent action of two antibiotics should be, and this precludes determining synergy or antagonism. Several models describe the independent action of two drugs, for example, Loewe additivity and Bliss independence. Baeder et al. [9] described a multi-hit model (following the idea of Hedges [24]) in which bacteria die when a given number of targets are “hit” by antimicrobials. Bliss independence assumes that there is no interaction between antimicrobials. While, Loewe additivity represents a measure of antimicrobials interaction, i.e., if there is synergy or antagonism. The authors suggest which model is best, based on the antimicrobials. In particular, Bliss independence is the best choice in the presence of different targets, while Loewe additivity is recommended when the two antimicrobials target the same component.

## Conclusions

Over the last 50 years, PK and PD modeling have shifted toward mechanistic approaches. In particular, the development of mechanistic PD modeling allows a deeper understanding of drug action, implying a broad array of future applications [45], such as antivirals, antibiotics, hypertension, inhibitors and any application that involves target binding. Here, we have discussed mathematical models describing molecular mechanisms of the drug action, and how these models can be applied to several diseases, which underlying assumptions we need and which phenomena can be captured. It is important to be aware of these assumptions.

For example in viruses, which neither replicate independently or can irreversibly die, the dynamics of the viral population can be neglected and viruses can be idealized as macromolecules with multiple binding sites [46]. Bacteria and cancer cells continuously divide and thereby reproduce the drug target. The chemically reversible processes (binding) are coupled to biologically irreversible processes (death). Depending on the speed of population turnover, a simplified model of a bacterium as a macromolecule will fail to adequately capture drug efficacy. It is also important to consider which parameters have been experimentally determined and to judge how much confidence one can have in those parameters. When chosing a more complex model, one should always be aware of the dangers of overparametrization if the parameters are not well known. However, when model and parameter choices match the biological systems, it has been repeatedly shown that both quantitative predictions, as well as biological understanding, can be vastly improved by the use of mechanistic models.
